# Assessment of volume status of pediatric hemodialysis patients

**DOI:** 10.1007/s00467-024-06409-2

**Published:** 2024-06-06

**Authors:** Fatina I. Fadel, Doaa M. Salah, Mohamed A. Abdel Mawla, Eman Galal, Shaimaa Sayed

**Affiliations:** 1 Pediatric department, Faculty of Medicine, Cairo University, 4 Extension of Nobar Street, Cairo, Egypt; 2 Pediatric department, National Research Center, Giza, Egypt

**Keywords:** Hemodialysis, Pediatrics, Fluid overload, Bioimpedance spectroscopy, Lung ultrasonography, Inferior vena cava

## Abstract

**Background:**

Accurate volume status assessment and dry weight achievement are the most challenging goals for a nephrologist. We aimed to evaluate the role of ultrasonographic parameters including lung ultrasound and inferior vena cava (IVC) measurements as practical methods of volume status assessment in children on hemodialysis by comparing them with established techniques, such as clinical evaluation and bioimpedance spectroscopy.

**Methods:**

A prospective cross-sectional study compared pre- and post-dialysis volume status using bioimpedance spectroscopy (BIS) parameters and clinical data with ultrasonographic lung B-lines and IVC parameters in children on regular hemodialysis.

**Results:**

A total 60 children (mean age 9.4 ± 2.8 years) were enrolled. Twenty patients (33.3%) were clinically overloaded to varying degrees (17 patients had mild to moderate signs of fluid overload and 3 patients had moderate to severe signs of fluid overload). All other patients (66.7%) were clinically euvolemic. Sonographic parameters were significantly lower post-dialysis than pre-dialysis, including lung B-line count and IVC diameter. IVC collapsibility index mean was significantly higher post-dialysis than pre-dialysis. There was a significant correlation between the lung B-line count, IVC parameters, and BIS-measured overhydration both before and after hemodialysis. Nine patients had ≥ 8 B-lines post-dialysis, only three of them were hypertensive.

**Conclusions:**

Clinical criteria alone are not specific for determining accurate fluid status in pediatric hemodialysis patients. Lung B-line score, IVC parameters, and BIS may be complementary to each other and to clinical data. Lung B-lines outperform IVC measurements and BIS in subclinical volume overload detection in pediatric hemodialysis patients.

**Graphical abstract:**

**Figure:**
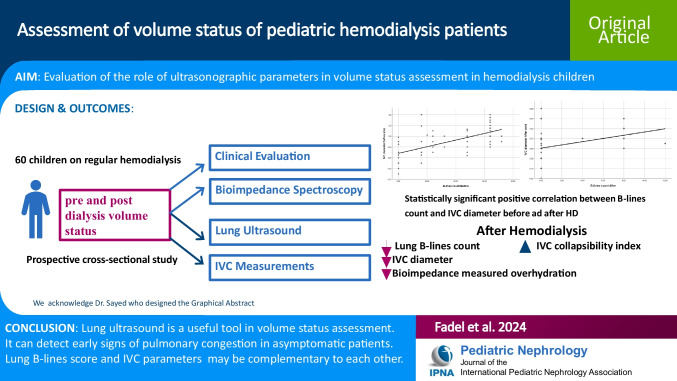
A higher resolution version of the Graphical abstract is available as Supplementary information

**Supplementary Information:**

The online version contains supplementary material available at 10.1007/s00467-024-06409-2.

## Introduction

One of the most crucial goals for children receiving kidney replacement therapy (KRT) for kidney failure is the success in achieving adequate fluid balance leading to true dry weight determination. Dry weight represents a clinically determined lowest post-dialysis body weight that the patient can tolerate without symptoms of hypovolemia [1, 2].

Chronic volume overload from inadequate fluid removal during dialysis can lead to increased cardiovascular morbidity. On the other side, excessive fluid removal might result in volume depletion, which can cause hypotension, cardiac stunning, and an earlier loss of residual kidney function [3].

Clinical assessments such as inter-dialytic weight gain (IDWG), the presence of hypotension or hypertension, and edema have traditionally been the mainstay of fluid assessment in pediatric hemodialysis (HD) patients. However, these clinical indicators are imprecise and insufficient to optimize the target dry weight [4].

Many methods exist to complete the clinical evaluation of the hydration status including echocardiography, inferior vena cava (IVC) parameters evaluated by ultrasound, biomarkers like brain natriuretic peptide, bioimpedance spectroscopy (BIS), plasmatic volume variation monitoring, and recently lung ultrasound (LUS) [5].

The use of LUS for the diagnosis of fluid overload in the dialysis population is receiving increasing attention due to its accuracy in measuring subclinical fluid overload, ability to detect changes in real time that allow for changing dialysis prescriptions, and ability to predict volume-related morbidity and mortality [6].

We aimed to evaluate the role of LUS and IVC parameters, as practical methods of volume status assessment in HD children by comparing them with established techniques such as clinical evaluation and BIS.

## Patients and methods

This is a prospective cross-sectional study, which was conducted at the Hemodialysis Section of Pediatric Nephrology Unit, Cairo University Children Hospital. The study included 60 children with chronic kidney disease (CKD) stage 5 on regular HD. This study has been approved by the Research Ethics Committee, Faculty of Medicine, Cairo University (approval code: D-38–2019).

Inclusion criteria: children aged 1–14 years and of both sexes presenting with CKD stage 5 on regular HD, three times per week for at least 3 months. Exclusion criteria: patients with congenital abnormalities of IVC (such as interruption of the hepatic segment of the IVC, azygos or hemiazygos continuation or duplication), patients with systemic diseases with vascular changes such as vasculitides and patients with parenchymal lung diseases or lung fibrosis.

After explaining the purpose of the study to the parents or guardians and taking informed consents from them, the volume status of each participant in the study was evaluated by the same pediatrician during a mid-week HD session using the following:

### History taking and clinical examination

Clinical history taking with special emphasis on the nature of original kidney disease, the start of KRT, and compliance with instructions regarding fluid intake.

- General examination including body weight and vital signs recording, heart rate (HR), respiratory rate (RR), and blood pressure (BP) measurements pre- and post-HD. BP measurements were performed by automated BP measurement in seated position.
- Clinical evaluation of over-hydration (OH) was calculated as follows: (pre-dialysis weight − dry weight)/dry weight, and expressed in percent. We estimated the dry weight clinically as the lowest post-dialysis body weight that the patient can tolerate without symptoms of hypovolemia.
- Evaluation of IDWG calculated as the average weight gain over the last three dialysis sessions.

### Laboratory data

Blood samples were obtained from each patient to assess the intradialytic change in the hematocrit by comparing the hematocrit level before and after HD.

### Bioimpedance spectroscopy analysis (BIS)

We assessed the water content using the multi-frequency BIS which is a non-invasive technique. The Fresenius body composition monitor (Fresenius Medical Care®), the most popular BIS device for children on HD, was used by our team. A total of 120 fluid assessments using body composition monitor were performed in the 60 children. Each child underwent two separate fluid assessments before and after the HD session. The body composition monitor measures at 50 frequencies over a range from 5 to 1000 kHz to determine the electrical resistances of the total body water (TBW) and the extracellular water (ECW). While high-frequency current passes through TBW, low-frequency current cannot penetrate cell membranes and thus flows exclusively through the extracellular water. The measurements included ECW, intracellular water (ICW), TBW, and over-hydration (OH), which were then estimated using equations in the device.

### Inferior vena cava ultrasound imaging

LOGIQ™ P7 ultrasound device with a 3Sc–RS phased array sector probe was used for IVC assessment by measuring the IVC diameter (IVCD) and collapsibility index (IVCCI) at end expiration, in supine position, measured within 1 cm from the junction between the IVC and suprahepatic veins. These assessments were performed within 30 min of starting the dialysis treatment and within 30 min of ending it. The IVCCI was calculated as follows: (maximum diameter on expiration (IVC max) − minimum diameter on inspiration (IVC min))/maximum diameter on expiration (IVC max).

### Lung ultrasound imaging

A portable ultrasound device (LOGIQ™ P7 with a 3–12 MHz linear probe) was used to conduct examinations at the patient’s bedside within 30 min of starting the dialysis treatment and within 30 min of ending it by the same operator. The scanning protocol was the eight-zone method, consisting of scanning four chest areas per side, to count the lung comets (B-lines). In each area, B-lines were quantified from 0 to 10 and a total B-line score was calculated. The normal total B-line score is from 0 up to 5 [7].

### Echocardiography

It was performed by a cardiologist. It was used to assess cardiac function and the presence of systolic dysfunction, diastolic dysfunction, and/or left ventricular hypertrophy.

### Statistical analysis

Data were coded and entered using the statistical package for the Social Sciences (SPSS) version 28 (IBM Corp., Armonk, NY, USA). Data was summarized using mean, standard deviation, median, minimum and maximum in quantitative data and using frequency (count) and relative frequency (percentage) for categorical data. Comparisons between groups were done using unpaired *t* test in normally distributed quantitative variables while non-parametric Mann–Whitney test was used for non-normally distributed quantitative variables***.*** For comparison of serial measurements (before and after) within each patient, paired *t* test was used in normally distributed quantitative variables, while non-parametric Wilcoxon signed rank test was used for non-normally distributed quantitative variables. Correlations between quantitative variables were done using Spearman correlation coefficient. *P* values less than 0.05 were considered as statistically significant.

## Results

**Fig. 1:**
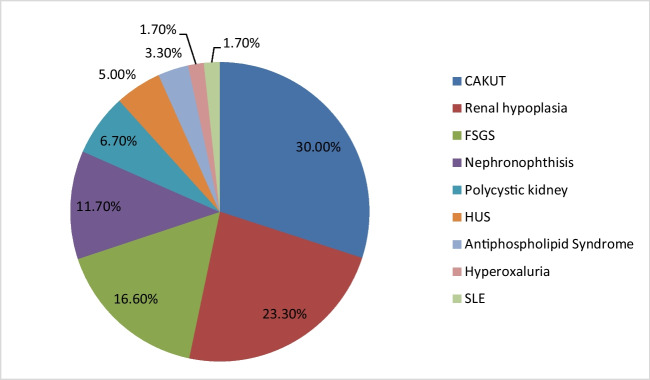
Primary renal disease of the study population

The current study included 60 children with CKD stage 5 on regular HD. Thirty-five patients were females (58.3%) and 25 patients were males (41.7%). Their mean ± SD age was 9.4 ± 2.8 years. The median age of onset of KRT was 3 years (range 0.5–8.0). Primary kidney disease of the study population is demonstrated in Fig. 1.

Forty children (66.7%) were compliant to fluid restriction instructions. Thirteen children (21.7%) were found to have cardiovascular disease, including diastolic dysfunction in 6 (10%), systolic dysfunction in 4 (6.7%), and left ventricular hypertrophy in 3 (5%) of them. In 47 children (78.3%), the cardiac function was normal. Forty-seven patients were hypertensive.

Regarding the clinical assessment of volume status before the HD session, we found that 20 patients (33.3%) had clinical signs of fluid volume overload. Seventeen patients had mild to moderate signs of fluid overload (10 had puffy eyelids, 7 had puffy eyelids and respiratory distress) and 3 patients had moderate to severe signs of fluid overload (puffy eyelids, respiratory distress and congested hepatomegaly), and the other patients (66.7%) were clinically euvolemic without any clinical signs of fluid volume overload. The patients in the current study did not have residual urine output. Clinical OH evaluation (%) median value was 5.45% (range 0.0–15.0). Median IDWG was 1 kg (range 0.2–2.6).

There were statistically significant lower mean values of TBW (L), ICW (L), ECW (L), OH (L), IVCD (cm), and B-line count after HD compared to before HD, with *P* < 0.001. Also, there were statistically significant higher mean values of IVCCI and hematocrit level after HD compared to before HD (*P* < 0.001) as demonstrated in Table 1. Nine of our patients (15%) experienced intradialytic hypotension (IDH) symptoms including vomiting, light-headedness, headache, abdominal pain, and sudden drop in BP.

**Table 1:** Comparison between bioimpedance parameters, IVC diameter, IVC collapsibility, B-line count, and hematocrit before and after HD session

	Mean	± SD	Median	Min	Max	*P* value
TBW (L)						
Before	15.07	4.09	15.00	7.40	25.40	< 0.001
After	14.16	3.87	13.75	6.90	24.30	
ICW (L)						
Before	8.31	2.27	8.15	4.30	14.00	< 0.001
After	8.13	2.23	8.10	4.20	13.90	
ECW (L)						
Before	6.78	1.88	6.85	3.00	11.40	< 0.001
After	6.03	1.71	5.95	2.70	10.40	
Overhydration (L)						
Before	0.55	0.61	0.40	− 1.30	2.00	< 0.001
After	− 0.12	0.36	− 0.10	− 1.70	0.90	
IVC diameter (cm)						
Before	0.88	0.25	0.90	0.30	1.40	< 0.001
After	0.42	0.15	0.40	0.10	0.80	
IVC Collapsibility (%)						
Before	43.08	7.66	45.00	25.00	60.00	< 0.001
After	58.70	6.26	60.00	45.00	70.00	
B-line count						
Before	16.30	12.90	14.00	0.00	36.00	< 0.001
After	1.20	2.97	0.00	0.00	12.00	
Hematocrit						
Before	33.01	4.44	33.25	21.40	43.00	< 0.001
After	34.39	4.46	34.35	23.50	43.20	

*TBW* total body water, *ICW* intracellular water, *ECW* extracellular water, *OH* overhydration, *IVC* inferior vena cava

A significant correlation was found between OH and other clinical parameters including, body weight, HR, RR, systolic BP (SBP), diastolic BP (DBP), IDWG, and clinical OH percentage before HD as demonstrated in Supplementary Material A.

**Fig. 2:**
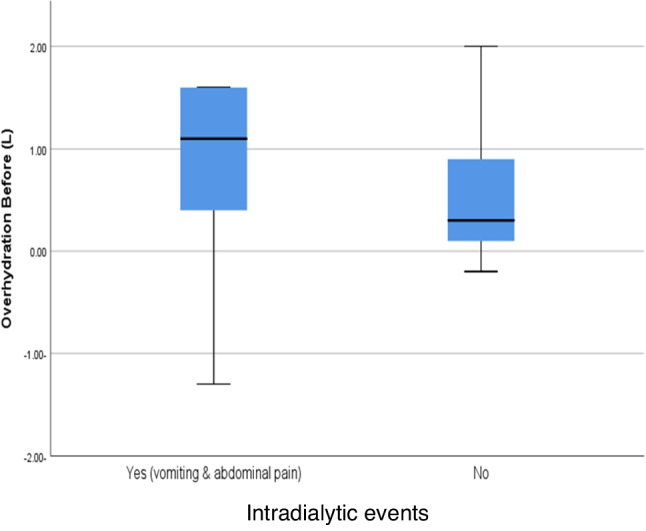
Box plot shows a statistically significant higher median value in intradialytic events than no intradialytic events, according to overhydration before hemodialysis (L), with *P* value = 0.044

According to OH (L) before HD, statistically significant higher median value of OH was found in children with intradialytic events than no intradialytic events with *P* = 0.044, Fig. 2.

**Fig. 3:**
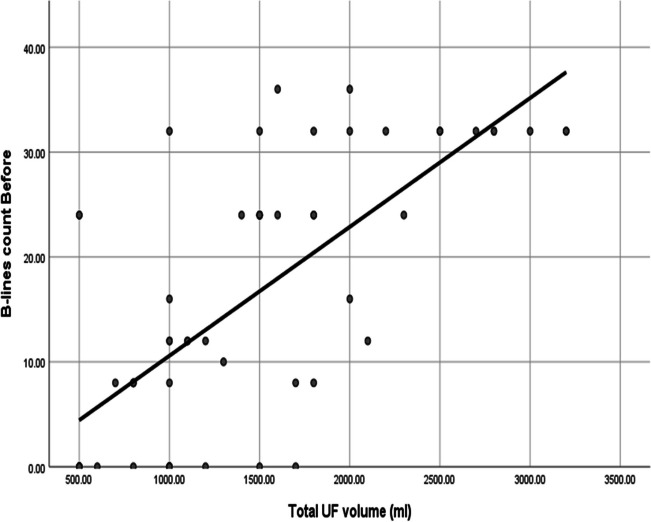
A significant positive correlation between B-line count and total UF volume before HD

**Fig. 4:**
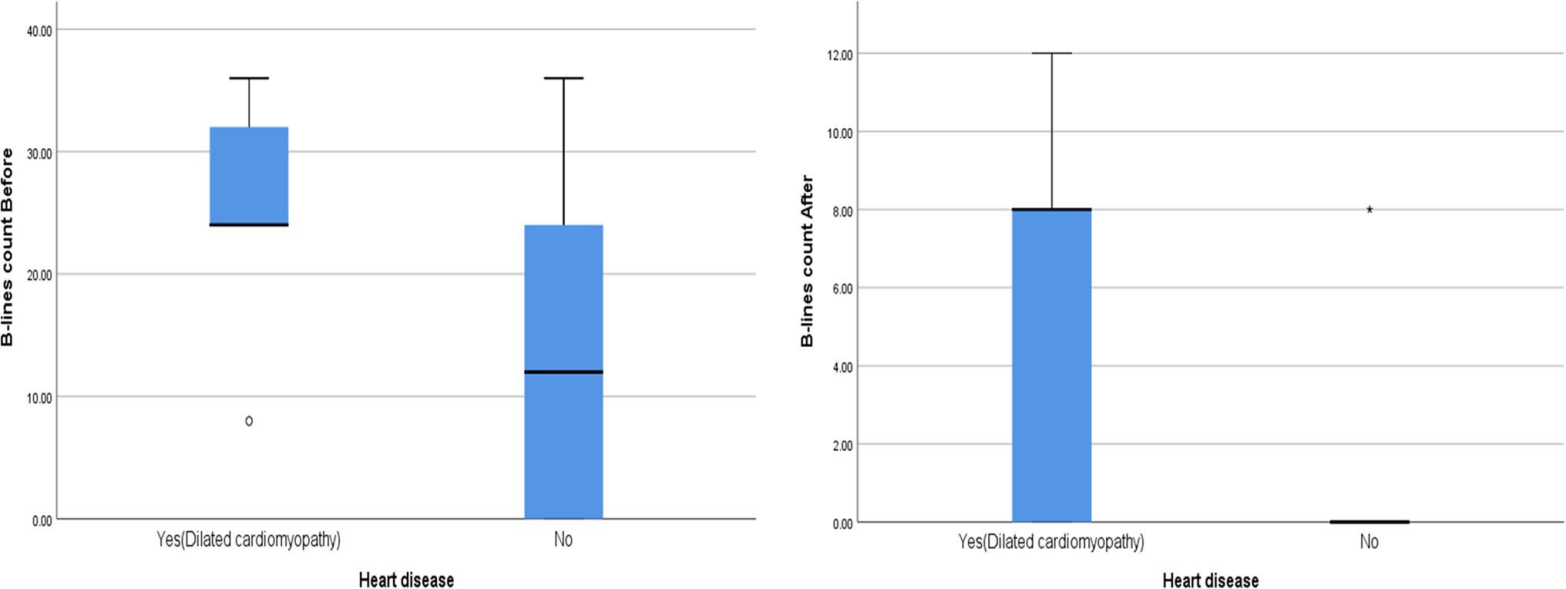
A statistically significant higher median value of B-line count before and after HD in children with cardiac disease

There was a significant correlation between B-line count and total ultrafiltration (UF) volume before the HD session as shown in Fig. 3, and a statistically significant higher median value of B-line count before and after HD in children with cardiac disease (Fig. 4).

There was a significant positive correlation between lung B-lines and other clinical parameters, including HR, RR, SBP, DBP, IDWG, and clinical OH percentage before the HD session, as demonstrated in Supplementary Material B.

Also, there was a significant positive correlation between the IVCD before HD and other clinical parameters including body weight, HR, SBP, DBP, IDWG, and clinical OH percentage. Additionally, a significant positive correlation was found between the IVCD and SBP, DBP after HD as demonstrated in Supplementary Material C. There was a significant negative correlation between the IVCCI before and after HD and the clinical OH percentage together with the IDWG as shown in Supplementary Material D.

There was a significant positive correlation between IVCD and B-line count before and after the HD session and a significant positive correlation between B-line count before HD and IVCD after HD. Also, there was a significant positive correlation between B-line count after HD and IVCD before HD. A significant negative correlation was found between IVCCI and B-line count before and after the HD session as demonstrated in Supplementary Material E.

**Fig. 5:**
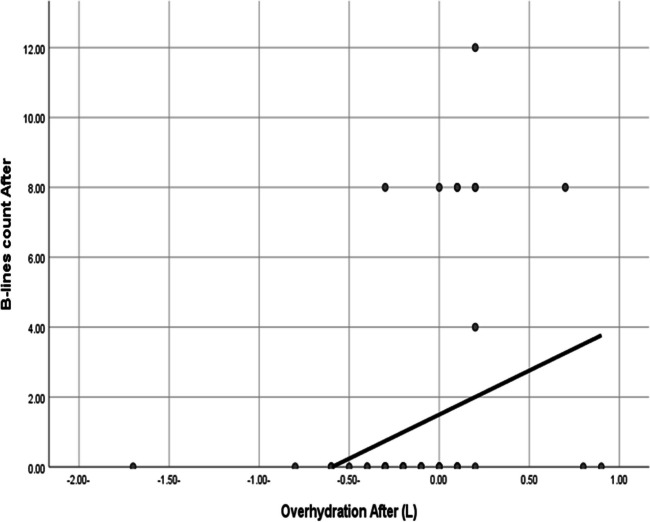
Statistically significant positive correlation between overhydration and B-line count after HD with *r* value = 0.423 and *P* < 0.001

As shown in Supplementary Material F, there was a significant correlation between the pre-dialysis OH and both IVC parameters (diameter and collapsibility), as well as B-line count before and after HD. Also, there was a significant positive correlation between post-HD OH and B-line score with (*r* value = 0.423 and *P* < 0.001) as demonstrated in Fig. 5.

## Discussion

Precise volume status assessment, permitting adequate post-dialysis weight and subsequent dry weight achievement, is one of the most challenging goals for a nephrologist. Both chronic volume overload and depletion lead to increased morbidity and mortality risk. Different methods are commonly used to assess the hydration status including clinical and vital signs, echocardiography, IVCD and IVCCI measurements, LUS, and BIS analysis [5]. Our aim was to evaluate the role of ultrasonographic assessment of extravascular fluid through lung B-line score, and intravascular fluid through IVC measurements, as practical tools for volume status assessment, and to compare them to conventional methods such as clinical evaluation and BIS.

The current study included 60 children; their age ranged from 3 to 14 years with mean of 9.45 ± 2.89 years. Twenty patients (33.3%) had clinical volume overload signs. All other patients (66.7%) were clinically euvolemic. These results are relatively comparable with a study by Allinovi et al. on 13 children on dialysis, where 82% of the children were assessed as clinically euvolemic, 14% as mildly dehydrated, and 4% as mildly overloaded. However, no children were assessed as having moderate or severe clinical fluid overload [8].

BIS has been presented as a useful, rapid, and non-invasive technique for assessing hydration status and body composition in HD patients. However, the inability to exchange devices and absence of a widely accepted method for interpreting hydration status from BIS measurements in pediatric dialysis patients make clinical application difficult [9]. In our study, each child’s volume status was assessed on two distinct occasions, including clinical examination and body composition monitor assessment before and after HD. All BIS measurements (TBW, ICW, ECW, and OH) had a statistically significant reduced mean value (*P* = 0.001) after HD, with the fluid removed from the patient largely originating from the ECW rather than the ICW. Similarly, other studies by Basso et al. and Youssef et al. compared various methods for fluid status assessment in patients on chronic HD and found the same results [10, 11].

On the other hand, Ehlayel et al. and Frey et al. found a statistically significant decrease in the mean ECW volume with a statistically significant increase in the mean ICW after HD [12, 13]. HD removes fluid mostly from the intravascular space. Thus, it is important to determine whether acute fluid removal alters the ECW and/or ICW compartments [13]. Zepeda-Orozco and Quigley explained the rise in ICW by the increase in the intracellular/extracellular osmotic gradient caused by urea elimination by dialysis [14].

In comparing BIS parameters with clinical data, our study found a strong relationship between pre-HD OH and other clinical parameters such as body weight, HR, RR, SBP, DBP, and clinical OH percentage. Also, Khin et al. showed a significant correlation between pre-dialysis SBP and ECW [15]. On the other hand, Zaloszyc et al. revealed that severe OH by BIS was found in 11.2% of HD sessions; however, the majority (73%) of that population had normal BP [16]. Zaloszyc et al. concluded that BP alone is insufficient for measuring hydration status in chronic dialysis patients. Accordingly, the generally held assumption that increased UF should benefit elevated BP to achieve a lower dry weight appears unsuitable, if not dangerous, especially in light of individual changes in hydration status over time. As a result, they advocated using body composition monitoring in body fluid measurement to support clinical assessment in pediatric dialysis patients with excessive hydration [16].

Also, fluid management using BIS compared to clinical estimation of hydration showed a decrease in mortality, relative fluid overload and BP with the use of BIS, as BIS provides an objective assessment of hydration [17, 18]. BIS analysis has only recently been utilized to determine the effect of OH on survival. Accordingly, our study discovered a strong correlation between OH before HD and intra-dialytic hypotension (IDH), particularly in patients with high IDWG. This is crucial because IDH is the most prevalent significant adverse event in pediatric HD, causing lasting multi-organ damage and increasing mortality.

Nine of our patients (15%) experienced IDH symptoms. When intolerance symptoms developed, all patients underwent passive leg rising and UF stop. Six patients received volume expansions of isotonic saline solution. Similarly, Torterüe et al. described IDH symptoms in 11 dialysis sessions (23%) [19]. According to a meta-analysis done by Wang and Gu, including 55 studies with 104,758 patients, BIS-OH parameters might be independent predictors for mortality and cardiovascular events in patients undergoing dialysis [20]. However, these studies were conducted on adult populations and did not have echocardiographic measures. Despite the significant intra- and interobserver variability, the prognostic value of different echocardiographic measures in patients on HD has been clearly demonstrated by Pinedo et al. [21].

The use of BIS during HD may aid in the prediction of cardiovascular compromise before the onset of symptoms and reduce the episodes of IDH due to changes in intravascular volume achieved with BCM-adjusted dry weight achievement [22, 23].

Recently, point-of-care ultrasound is being used more often in a number of clinical settings to enhance the evaluation of patients’ fluid status owing to its non-invasive nature and relative ease and portability. Sonographic parameters were assessed in addition to clinical parameters, both pre- and post-dialysis for all participants in our study. We found that there was a significant reduction in all clinical parameters (body weight, HR, RR, SBP, DBP), as well as sonographic parameters (total B-line number and IVC max diameter) after HD. This is consistent with the findings of Haskin et al., who found that clinical parameters (weight, SBP, DBP) as well as sonographic data (total B-line number and max IVCD) were significantly lower post- than pre-dialysis [24]. Ehlayel et al. and Youssef et al. also reported that there was a statistically significant difference in the expiratory IVCD and IVCCI, and B-line count before and after dialysis [7, 12]. Also, other studies found that all patients had significant decrease in B-line scores after dialysis [8, 25, 26].

However, patients with interstitial lung diseases and lung fibrosis were excluded from our study since B-lines may present in these circumstances, making it impossible to determine if these B-lines are related to volume overload or other lung pathologies, which was also concluded by Martelius et al. [27].

Several studies have employed lung B-lines to determine extravascular volume. Nine of our patients had ≥ 8 B-lines at the end of dialysis, indicating fluid overload. Only three of them were hypertensive, implying that sonographic scanning can help detect covert fluids in normotensive patients. Also, the existence of lung B-lines in asymptomatic patients demonstrates the capacity of LUS to detect early signs of pulmonary congestion. Similarly, Youssef et al. and Vareesangthip et al. concluded that LUS is a useful tool to detect subclinical pulmonary congestion [7, 28].

Furthermore, Razaq et al. found that LUS revealed pulmonary congestion in 62.9% of patients, while clinical evaluation only detected it in 30.8% of patients [29]. According to Siriopol et al., subclinical volume overload is an independent predictor of death and cardiac events in adult patients on HD. Furthermore, they concluded that the LUS B-line score was the best predictor for the relationship between hydration status and mortality, independently of BIS-derived parameters [30].

Our study found a significant correlation between B-line count and impaired myocardial contractility before and after HD with *P* value < 0.001. Cardiac disease was identified in 8 of the 9 patients who still had B-lines at the end of dialysis; five of them had diastolic dysfunction while the other three had systolic dysfunction. This is in concordance with the randomized controlled study done by Loutradis et al. evaluating LUS-guided dry weight reduction monitoring, which found that patients with a history of cardiovascular disease had a higher B-line score than patients without a history of cardiovascular disease. This shows that when utilizing LUS to determine volume status, we should consider the effect of heart disease on the B-line score [6]. Another adult study was conducted by Youssef et al., which found a statistically significant negative correlation between lung B-lines and ejection fraction [11].

In a multicenter study with 392 HD patients, Zoccali et al. found that the high incidence of pulmonary congestion in both symptomatic and asymptomatic patients was closely linked with left ventricular ejection fraction. They tested the predictive significance of extravascular lung water as determined by LUS. When compared to patients with mild or no pulmonary congestion, those with extremely severe pulmonary congestion had a higher risk of cardiovascular events and death. This strong association between these echocardiographic measures and lung B-lines implies that left ventricular dysfunction contributes significantly to lung congestion in patients on HD [2].

Among our 60 patients, 120 lung ultrasound assessments were performed with two separate assessments for each patient before and after HD. There was a significant correlation between the B-line score and the total UF volume. Also, other studies found that the change in B-line number correlated positively with UF volume and weight change during dialysis; signifying that the reduction of B-lines was in proportion to the UF volume [8, 24, 25].

LUS can be a reliable predictor of a patient’s volume status and it is inexpensive, rapid to conduct, relatively simple to learn and can be done at the bedside. Another benefit is that LUS measures changes in real time, making it more practical because it may be used during or soon after dialysis. It can help dialysis prescription changes, such as prolonging the length of the dialysis session or increasing the volume of UF to improve volume status if it is done minutes before the end of the dialysis session especially in high-risk patients. Thus, it can be a valuable method for monitoring dry weight in pediatric patients on HD [25].

We tried to evaluate the role of IVC parameters in pediatric patients on HD. IVCD and IVCCI are sonographic parameters that change in accordance with intravascular volume status, as central venous pressure (CVP) has a positive correlation with IVCD and a negative correlation with IVCCI. In our study, there were significant decreases in IVCD post-dialysis measurements. Indeed, a study conducted by Muniz Pazeli et al. found that IVCD decreased after dialysis in children [31].

We found also that there was a statistically significant higher mean value of IVCCI after HD. These results are similar to those of Arun Thomas et al., who reported a significant increase in IVCCI after dialysis [32]. Furthermore, Youssef et al. found a statistically significant difference between the IVCD and IVCCI before and after HD [7].

Our study showed a significant correlation between the pre-dialysis OH and both IVC parameters (diameter and collapsibility). Similarly, Allinovi et al. reported comparable results on 22 IVC measures in 13 patients on dialysis, to BIS hydration status. The partitioning of extravascular volume between the interstitial and intravascular compartments could explain these findings. Despite being an excellent predictor of CVP, IVC measures are poor predictors of extracellular hydration. Thus, changes in IVC width and IVCCI during dialysis sessions may simply be a sign of intravascular volume depletion [8].

On studying the correlation between IVC parameters and other clinical data, we found a statistically significant positive correlation between the pre-dialysis IVCD and other clinical parameters including volume overload signs, body weight, SBP, and DBP.

In the current study, we found a statistically significant positive correlation between clinical volume overload signs and each of the BIS-OH, B-line count, and IVCD, as well as a statistically significant negative correlation with IVCCI before and after HD. We also found that there was a statistically significant negative correlation between B-lines and IVCCI before and after HD. Similarly, Gohary et al. found the same results [33]. Comparing different methods together, we found that B-lines were significantly reduced after HD. This reduction was significantly related to BIS-OH and IVC parameters.

Other studies showed that all sonographic parameters including total B-lines, IVCD, and IVCCI significantly changed during dialysis. Also, they concluded that lung B-lines are superior to echocardiography and BIS in detecting volume overload in patients on dialysis [24, 30]. However, they concluded that assessing both the B-line count and IVC parameters lends weight to sonographic findings when both parameters point to the same fluid status conclusion. Also, Ahmed et al. concluded that the combination of lung and IVCCI ultrasonography could be a valuable method for monitoring dry weight in pediatric patients on dialysis [34].

However, there is a wide variation in the absolute values of IVC diameter with age in the pediatric population. Also, there are no standard normal values for IVCD, and significant inter-individual variations necessitate serial measurements for each patient in order to determine each patient’s normal IVCD. Hence, relaying absolute IVCD for volume status assessment is difficult and cannot be used as a single parameter for fluid status assessment [35, 36].

The current study showed a statistically significant higher mean value of hematocrit level after HD, which can be used as an indirect volume status indicator. Also, Pstras et al. found a statistically significant increase in the hematocrit level after HD [37].

To conclude, conventional approaches based on clinical criteria alone are not always accurate and are not specific for determining accurate fluid status in pediatric patients on HD. Similarly, volume status cannot be assessed by IVC indices independently. However, these patients can benefit from non-invasive techniques like ultrasonographic B-lines and IVC parameters for a better dry weight achievement and better management of their hemodynamics. These techniques support one another and the clinical data.

LUS is a useful tool in volume status assessment, being the most practical of the three methods, owing to its advantages, and we recommend its routine use in the assessment and management of pediatric HD patients. Concomitant use of BIS may be needed with LUS for more accurate assessment of volume status in patients on HD.

The present study is one of the few that uses a single modality for extravascular and intravascular fluid status via performance of ultrasonographic assessment of lung B-lines and IVC parameters, in comparison with BIS for volume status assessment in pediatrics. Moreover, all examinations were performed by one researcher, and this enables a single operator to perform a bedside procedure that is quick and simple. To the best of our knowledge, this is one of the largest single-center studies evaluating volume status in children on regular HD.

Another strength point is repeated measures in each participant, where each child underwent two separate full assessments including clinical data, BIS, and LUS assessment of lung B-lines and IVC parameters before and after HD.

### Limitations

This study is mono-centric study. The lack of “gold standard” evaluation of hydration status such as deuterium dilution is not actually available in clinical settings. Another limitation is intrinsic to the ultrasound method, being operator-dependent.

### Supplementary Information

Below is the link to the electronic supplementary material.

Graphical abstract (PPTX 84.0 KB)

Supplementary file2 (DOCX 660 KB)

## Data Availability

The datasets used and/or analyzed during the current study are available from the corresponding author on reasonable request.
