# Supplementary material for: Assessment of volume status of pediatric hemodialysis patients
Source: Pediatr Nephrol. 2024 Jun 6;39(10):3057–66. doi: 10.1007/s00467-024-06409-2 (PMC11349778; doi:10.1007/s00467-024-06409-2)
Supplement: Supplementary file 1 — Graphical abstract (PPTX 84.0 KB) [file 467_2024_6409_MOESM1_ESM.pptx]

## Slide 1
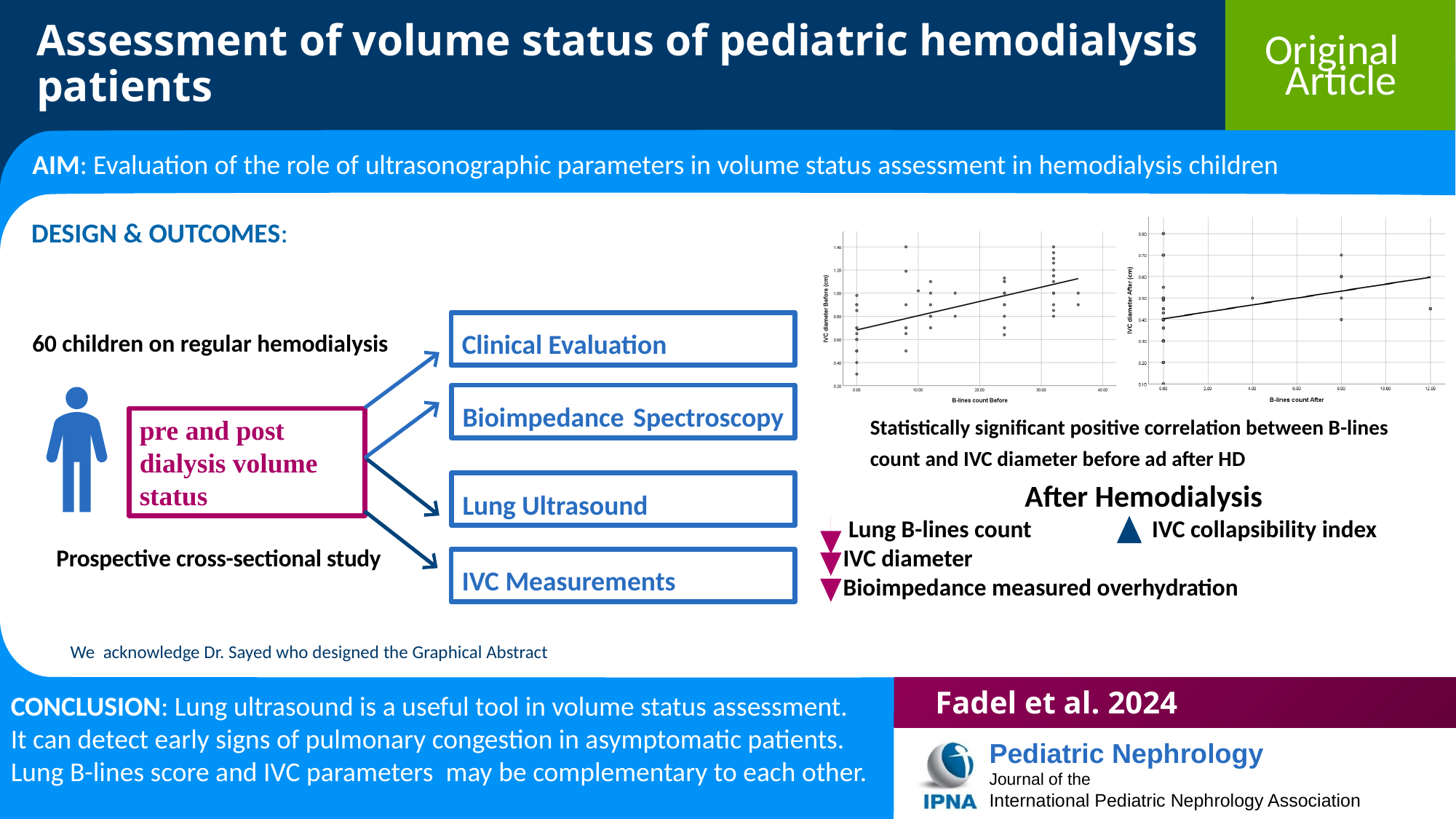

Assessment of volume status of pediatric hemodialysis patients
AIM: Evaluation of the role of ultrasonographic parameters in volume status assessment in hemodialysis children
DESIGN & OUTCOMES:
Clinical Evaluation
60 children on regular hemodialysis
Bioimpedance Spectroscopy
pre and post dialysis volume status
Statistically significant positive correlation between B-lines count and IVC diameter before ad after HD
After Hemodialysis
 Lung B-lines count IVC collapsibility index
IVC diameter
Bioimpedance measured overhydration
Lung Ultrasound
Prospective cross-sectional study
IVC Measurements
We acknowledge Dr. Sayed who designed the Graphical Abstract
 Fadel et al. 2024
CONCLUSION: Lung ultrasound is a useful tool in volume status assessment. It can detect early signs of pulmonary congestion in asymptomatic patients. Lung B-lines score and IVC parameters may be complementary to each other.
