# Supplementary material for: Assessment of volume status of pediatric hemodialysis patients
Source: Pediatr Nephrol. 2024 Jun 6;39(10):3057–66. doi: 10.1007/s00467-024-06409-2 (PMC11349778; doi:10.1007/s00467-024-06409-2)
Supplement: Supplementary file 2 — Supplementary file2 (DOCX 660 KB) [file 467_2024_6409_MOESM2_ESM.docx]

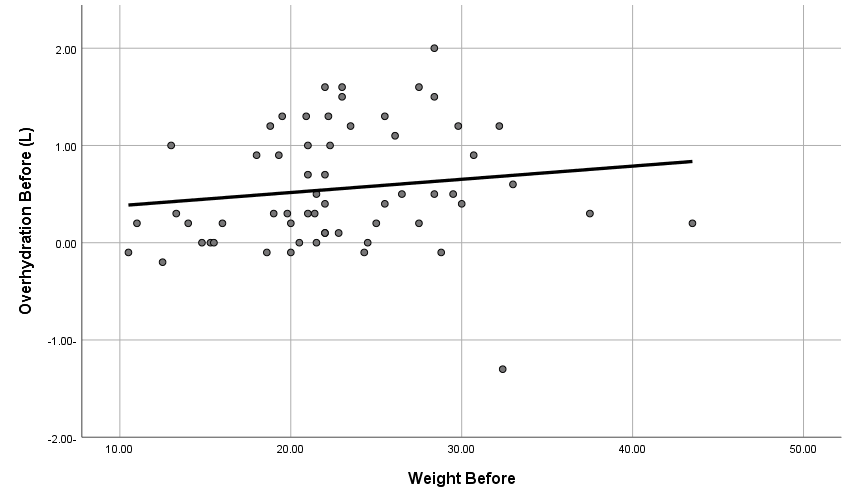

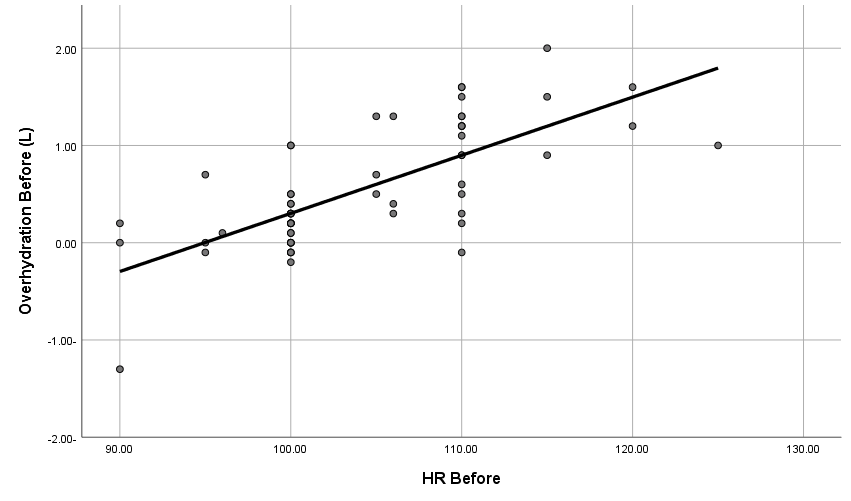


| **(a):** Statistically significant positive correlation between weight and over hydration value before HD with (r-value = 0.273 and p=0.035). | **(b):** Statistically significant positive correlation between HR and over hydration value before HD with (r-value = 0.712 and p<0.001). |
| --- | --- |
| 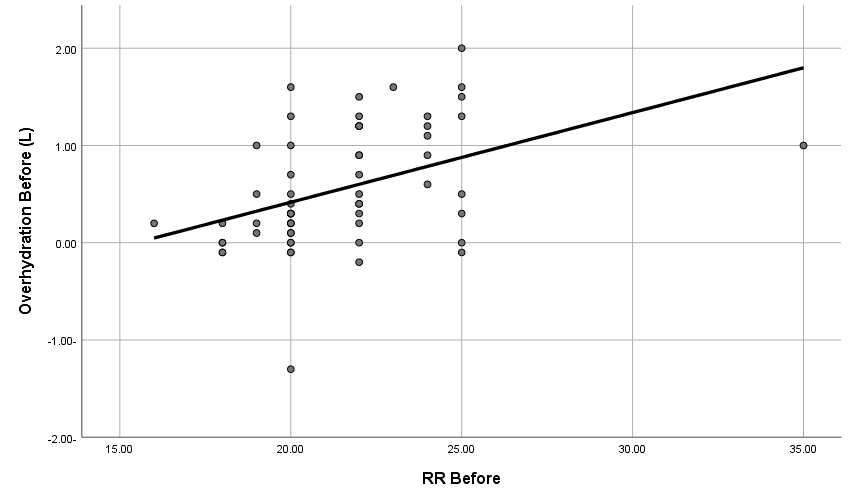 | 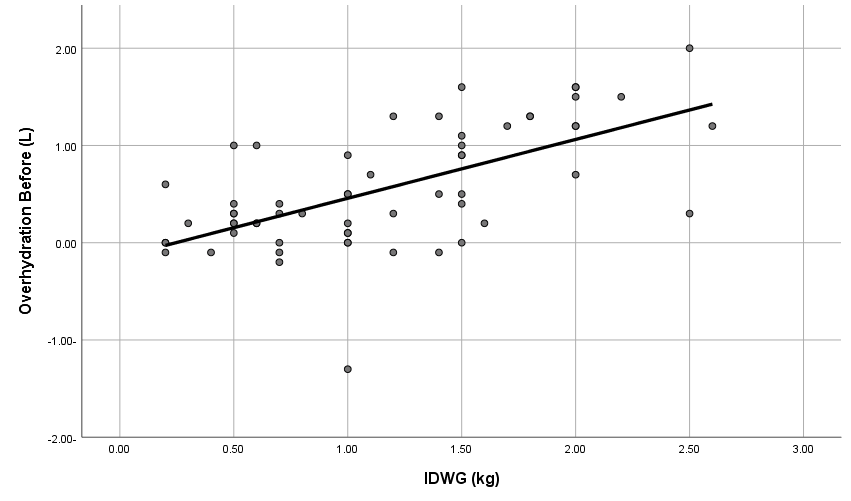 |
| **(c):** Statistically significant positive correlation between RR and over hydration value before HD with (r-value = 0.493 and p<0.001). | **(d):** Statistically significant positive correlation between IDWG (kg) and over hydration value before HD with (r-value=0.616 and p<0.001). |
| 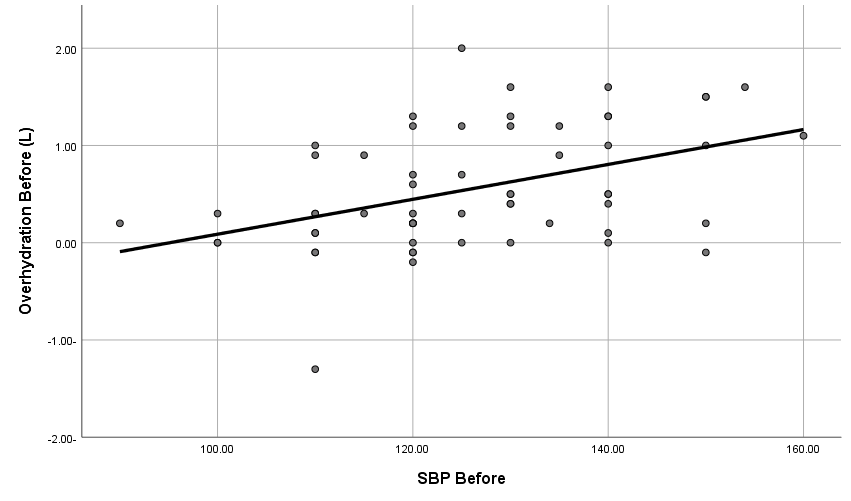 | 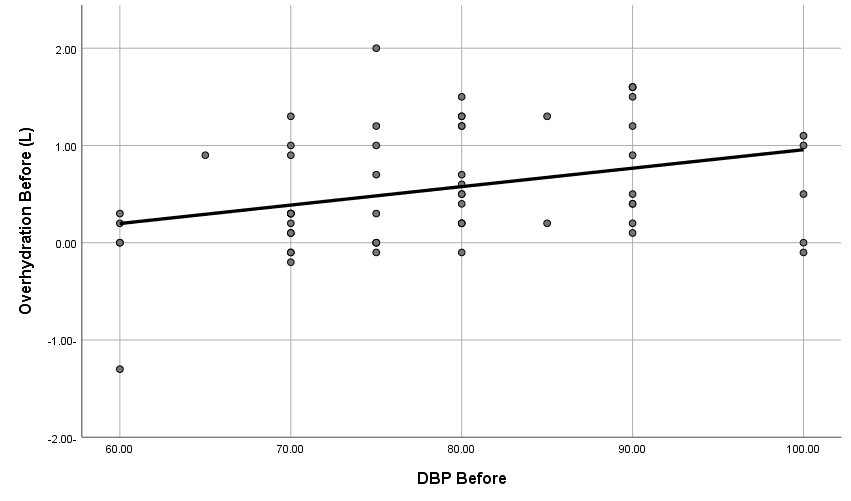 |
| **(e):** Statistically significant positive correlation between SBP and over hydration value before HD with (r-value = 0.446 and p<0.001). | **(f):** Statistically significant positive correlation between DBP and over hydration value before HD with (r-value = 0.350 and p=0.006). |
| 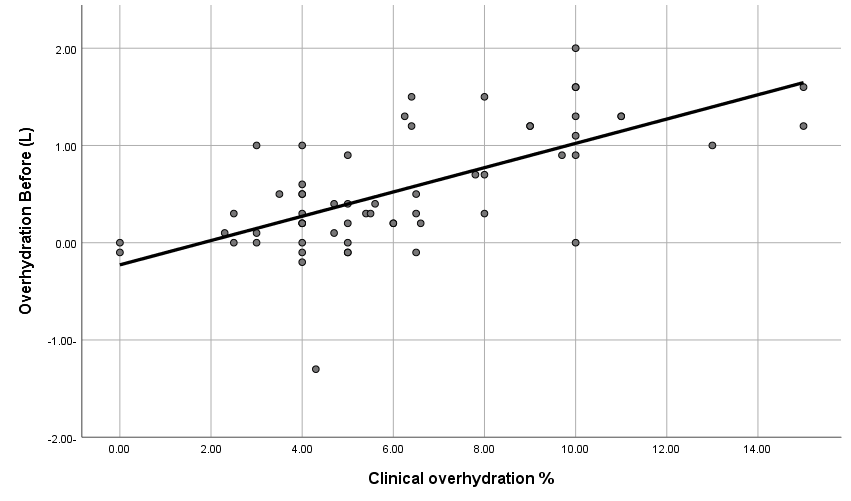 | |
| **(g):** Statistically significant positive correlation between clinical over hydration% and over hydration value before HD with (r-value = 0.628 and p<0.001). | |

**Supp. A: a significant correlation between OH and other clinical parameters including, weight, HR, RR, SBP, DBP, IDWG and clinical overhydration percentage before HD.**

| 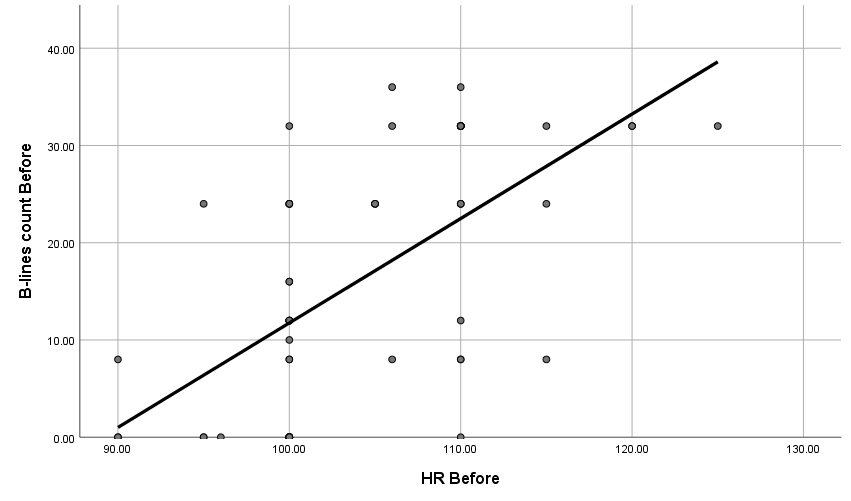 | 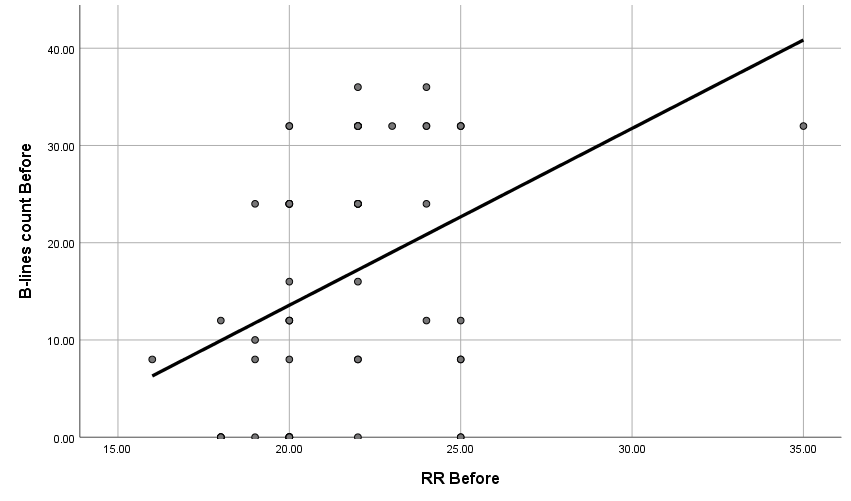 |
| --- | --- |
| **(a):** Statistically significant positive correlation between HR and B-lines count before HD with (r-value = 0.360 and p<0.001). | **(b):** Statistically significant positive correlation between RR and B-lines count before HD with (r-value = 0.453- and p<0.001). |
| 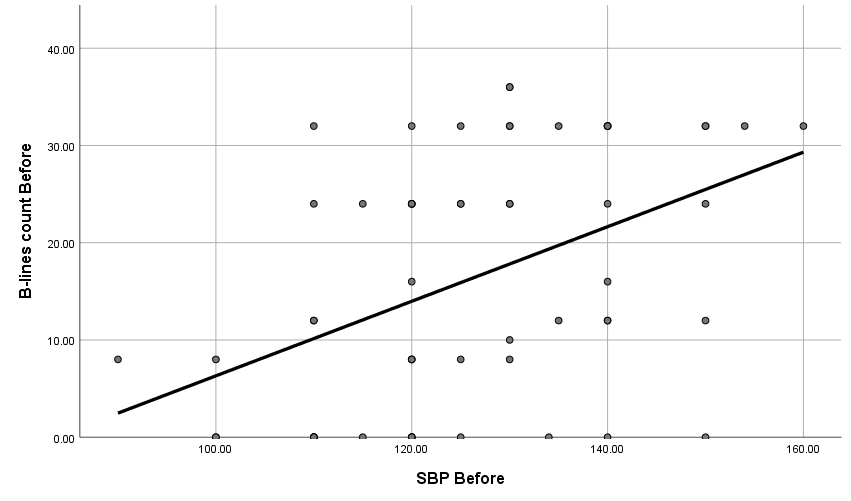 | 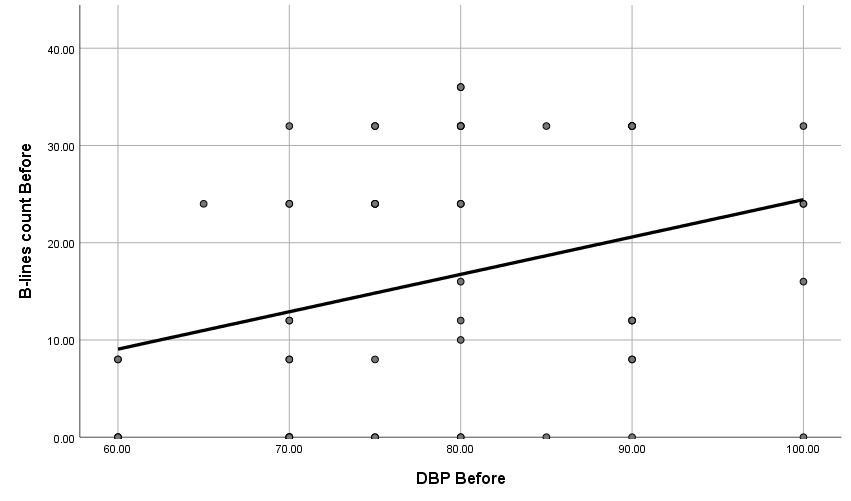 |
| **(c):** Statistically significant positive correlation between SBP and B-lines count before HD with (r-value = 0.470 and p<0.001). | **(d):** Statistically significant positive correlation between DBP and B-lines count before HD with (r-value = 0.360- and p<0.005). |
| 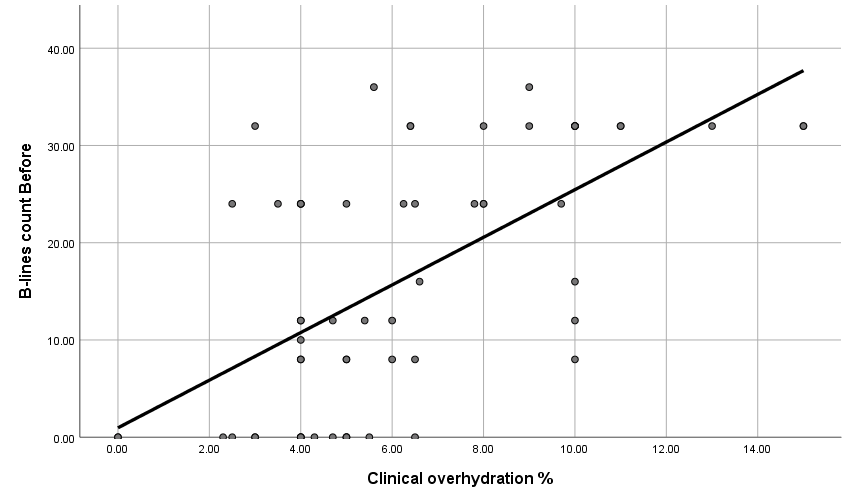 | 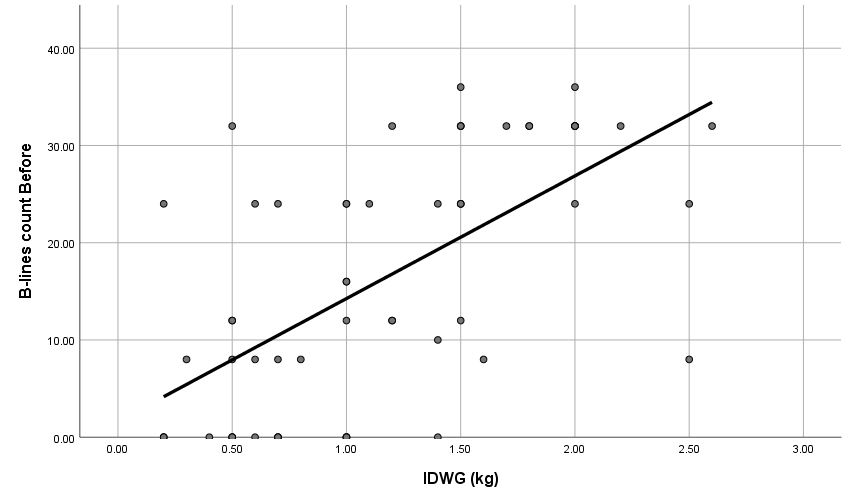 |
| **(e):** Statistically significant positive correlation between Clinical overhydration % and B-lines count before HD with (r-value = 0.602 and p<0.001). | **(f):** Statistically significant positive correlation between lDWG (Kg) and B-lines count before HD with (r-value = 0.649- and p<0.001). |

**Supp. B:** Correlations between lung B-lines and other clinical parameters including, HR, RR, SBP, DBP, IDWG and clinical overhydration percentage before the HD session

| 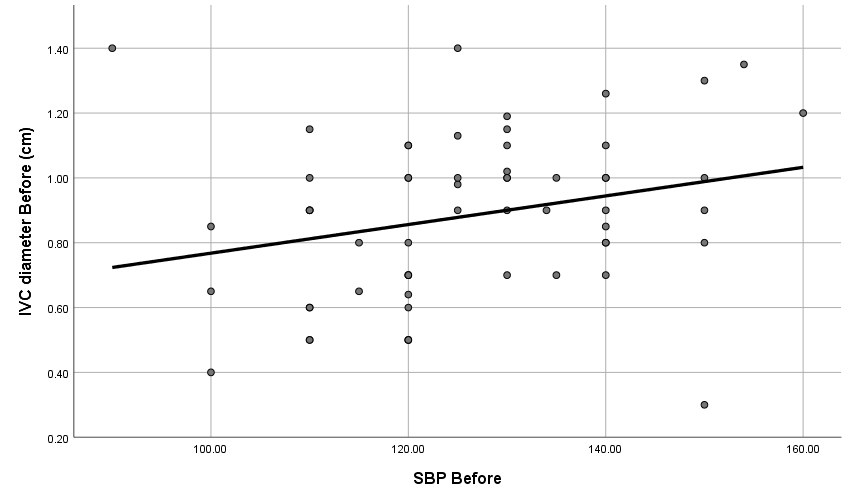 | 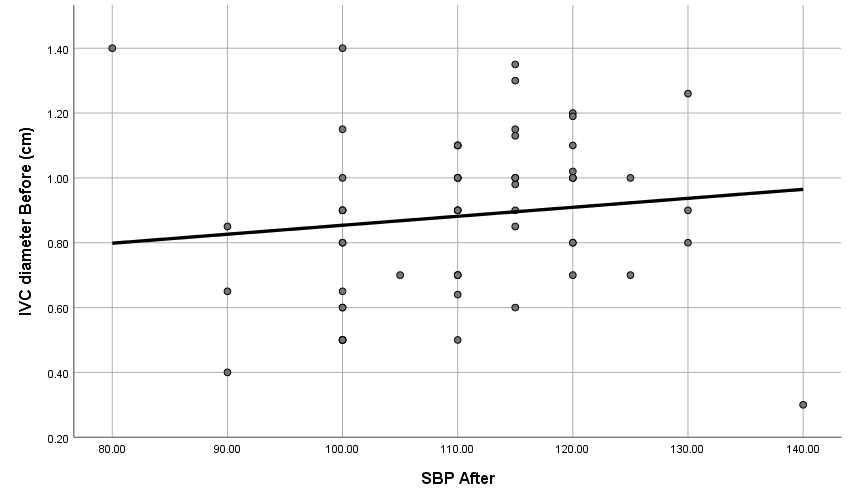 |
| --- | --- |
| **(a):** Statistically significant positive correlation between SBP and IVC diameter before HD  with (r-value = 0.328 and p=0.010). | **(b):** Statistically significant positive correlation between IVC diameter before HD and SBP after HD with (r-value = 0.273 and p=0.035). |
| 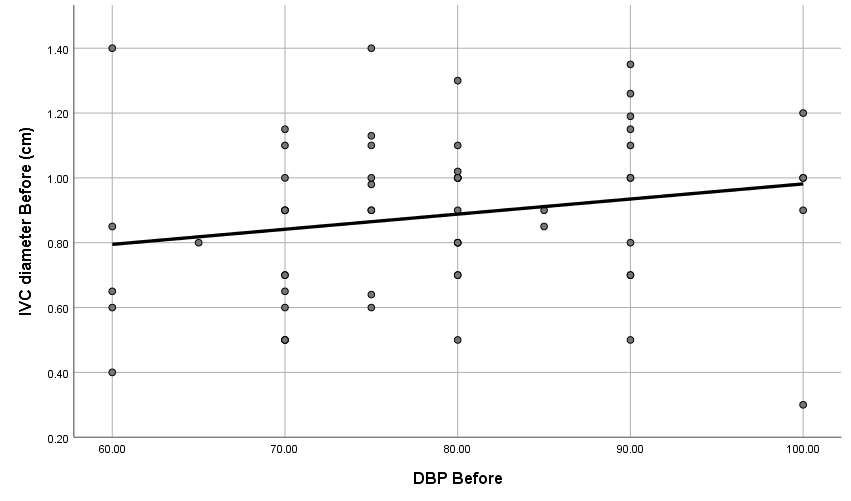 | 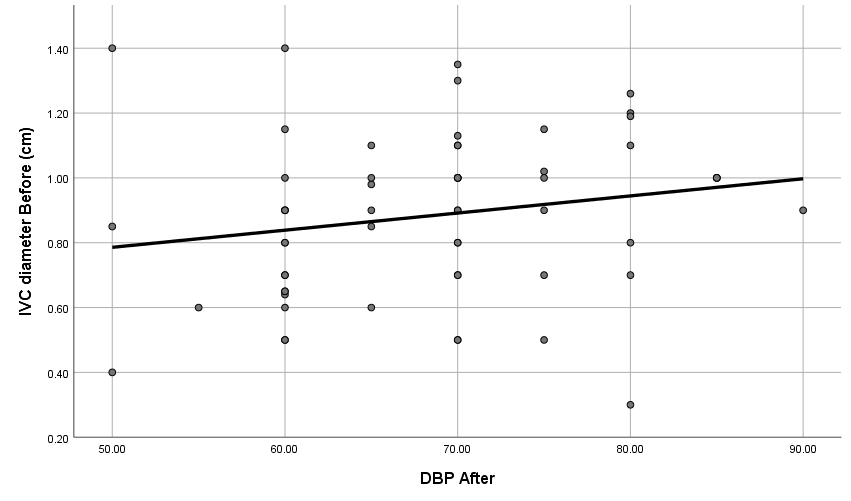 |
| **(c):** Statistically significant positive correlation between DBP and IVC diameter before HD  with (r-value = 0.267 and p=0.039). | **(d):** Statistically significant positive correlation between IVC diameter before HD and DBP after HD with (r-value = 0.272 and p=0.036). |
| 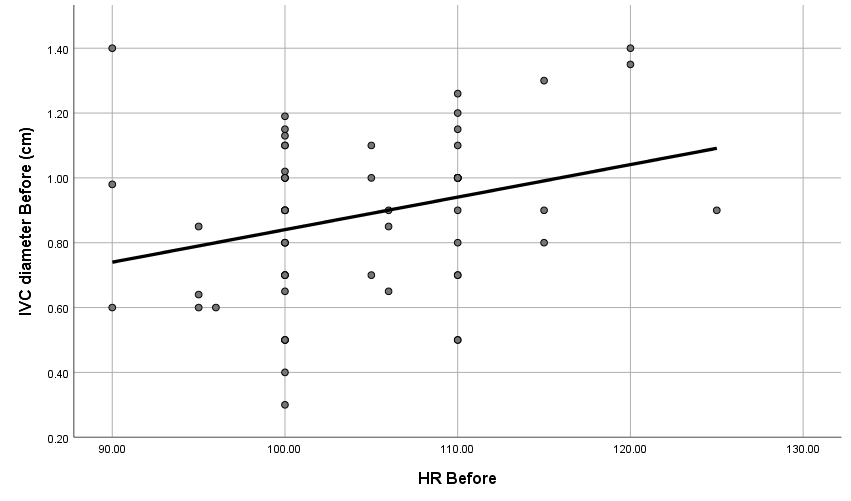 | 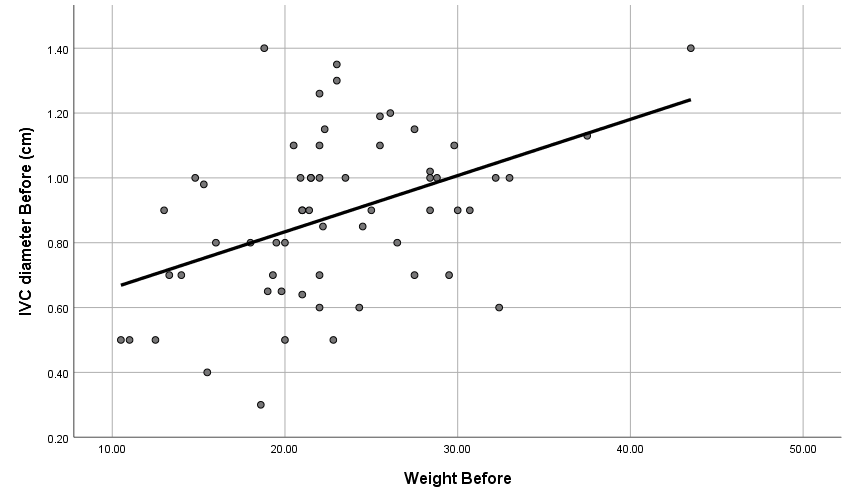 |
| **(e):** Statistically significant positive correlation between HR and IVC diameter before HD  with (r-value = 0.295 and p=0.022). | **(f):** Statistically significant positive correlation between weight and IVC diameter before HD  with (r-value = 0.432 and p=0.001). |
| 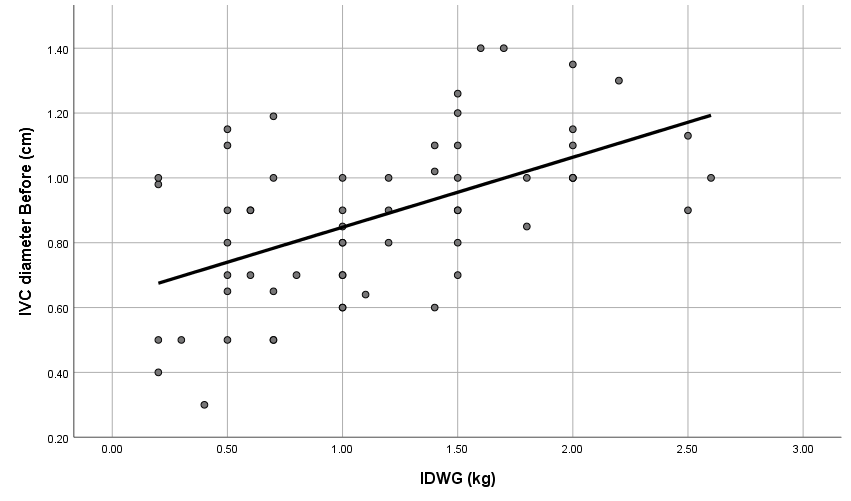 | 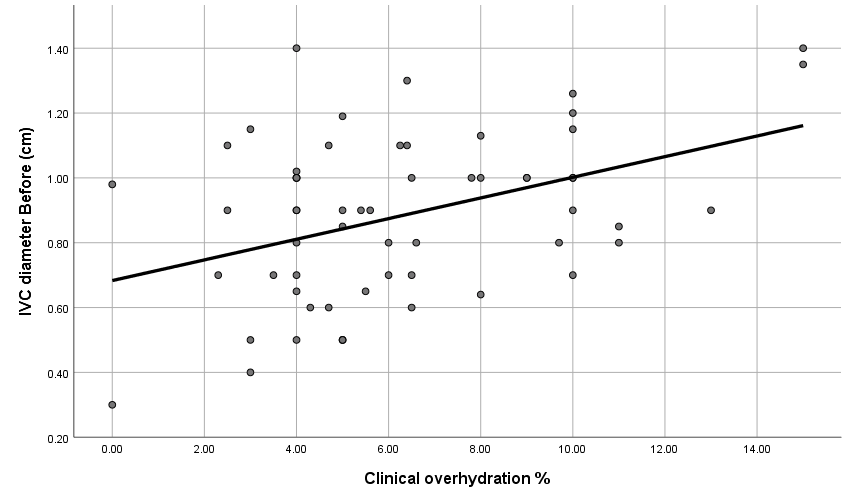 |
| **(g):** Statistically significant positive correlation between IDWG (kg) and IVC diameter before HD with (r-value = 0.542 and p<0.001). | **(h):** Statistically significant positive correlation between Clinical overhydration% and IVC diameter before HD with (r-value = 0.302 and p=0.019). |

**Supp. C: correlation between the IVC diameter Before HD and other clinical parameters including, body weight, HR, SBP, DBP, IDWG and clinical overhydration percentage**

| 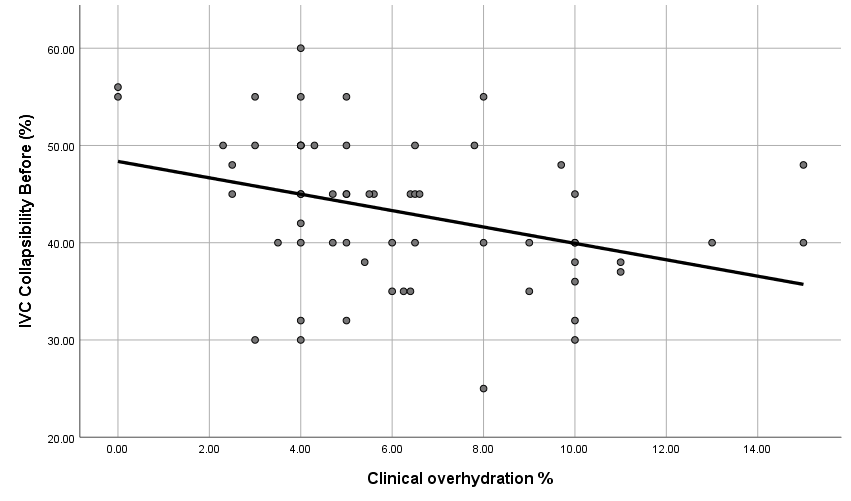 | 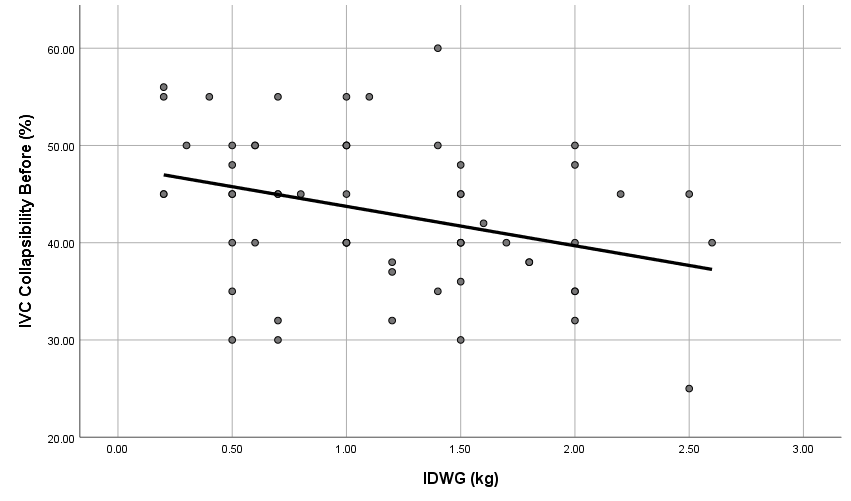 |
| --- | --- |
| **(a):** Statistically significant negative correlation between Clinical overhydration % and IVCCI before HD (with (r-value -0.406 and p<0.001). | **(b):** Statistically significant negative correlation between IDWG (kg) and IVCCI before HD with (r-value -0.348 and p<0.006). |
| 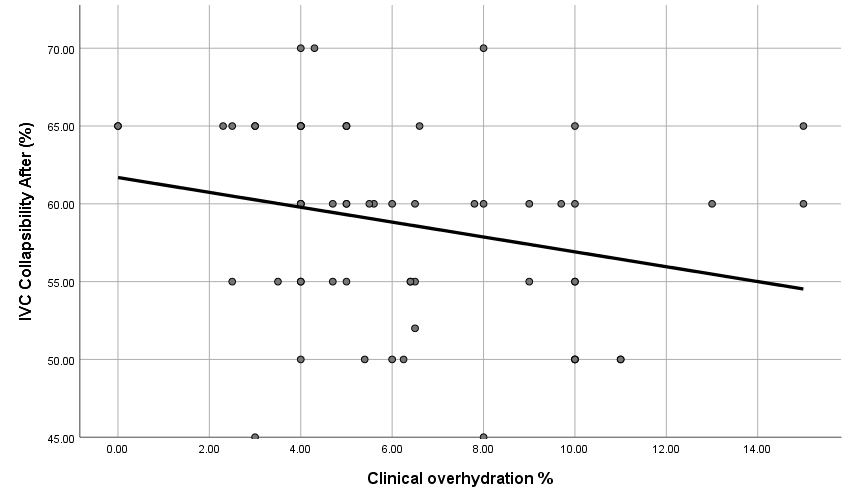 | 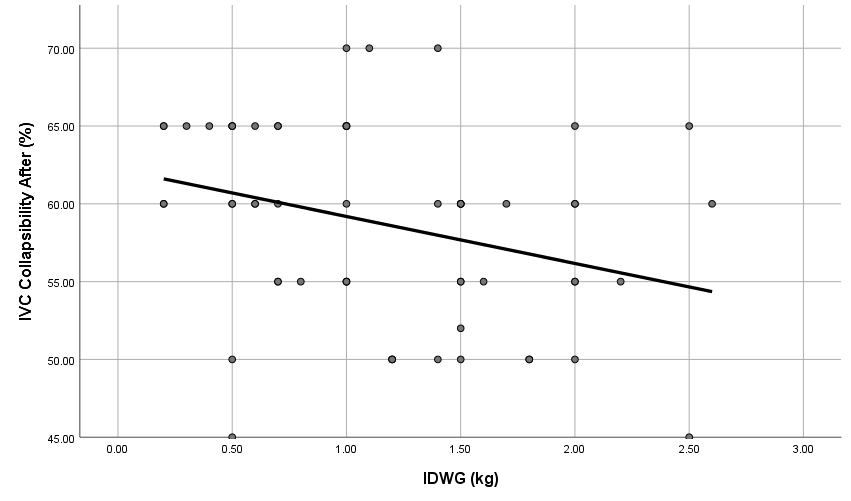 |
| **(c):** Statistically significant negative correlation between Clinical overhydration % and IVCCI after HD with (r-value -0.324 and p<0.012). | **(d):** Statistically significant negative correlation between IDWG (kg) and IVCCI after HD with (r-value -0.344 and p<0.007). |

**Supp. D: correlation between the IVC collapsibility index after HD and the clinical overhydration percentage together with the IDWG**

| 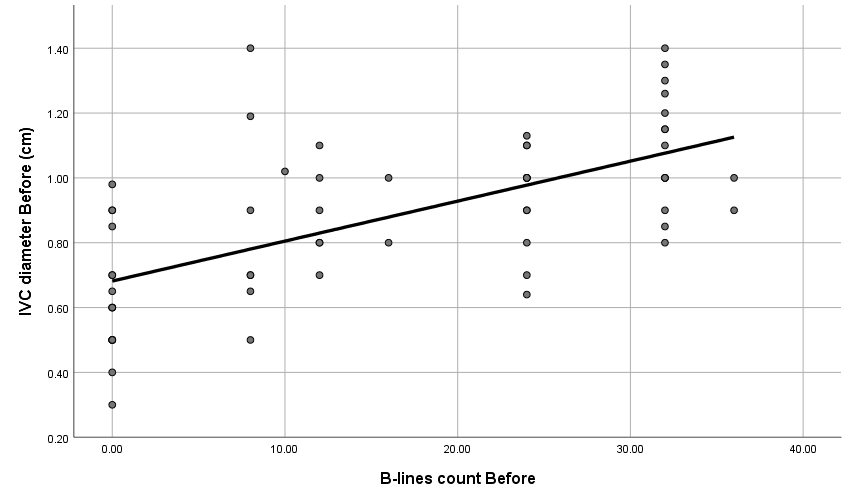 | 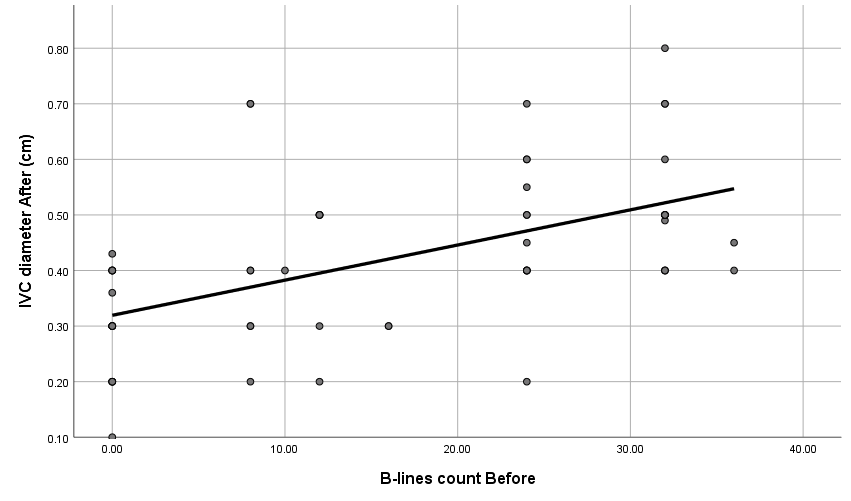 |
| --- | --- |
| **(a):** Statistically significant positive correlation between B-lines count and IVC diameter before HD  with (r-value = 0.641 and p<0.001). | **(b):** Statistically significant positive correlation between B-lines count before and IVC diameter after HD  with (r-value = 0.572 and p<0.001). |
| 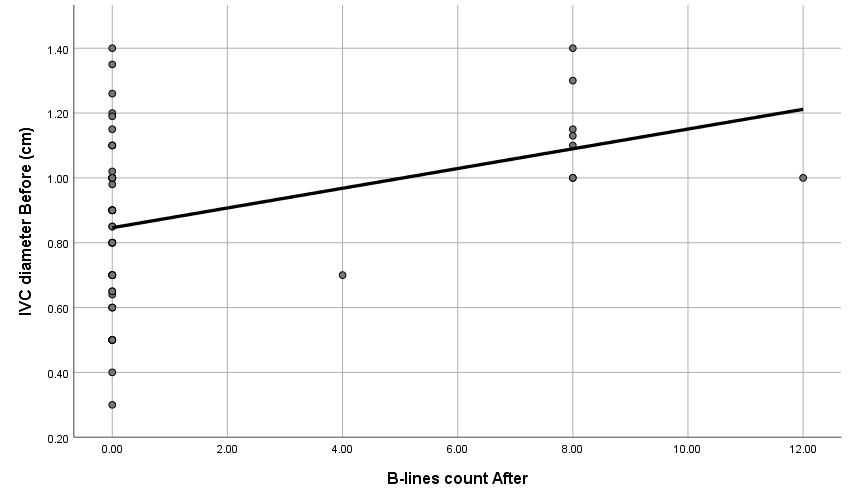 | 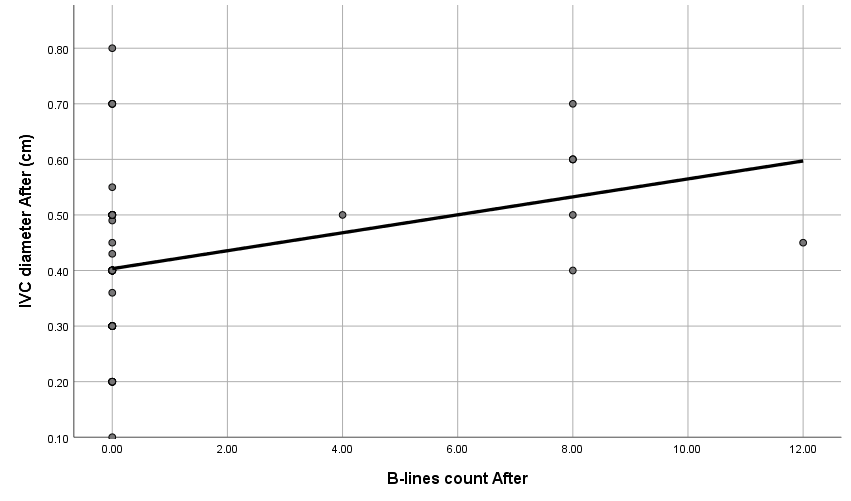 |
| **(c):** Statistically significant positive correlation between B-lines count after and IVC diameter before HD  with (r-value = 0.365 and p=0.004). | **(d):** Statistically significant positive correlation between B-lines count and IVC diameter after HD  with (r-value = 0.361 and p=0.005). |
| 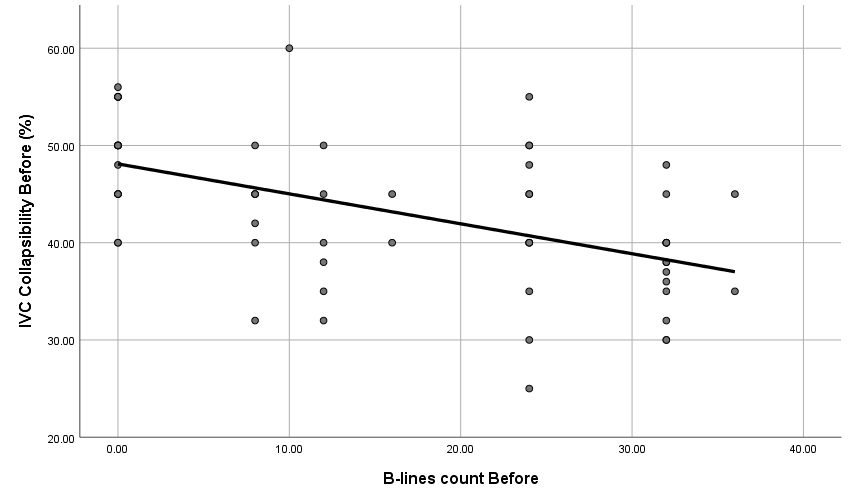 | 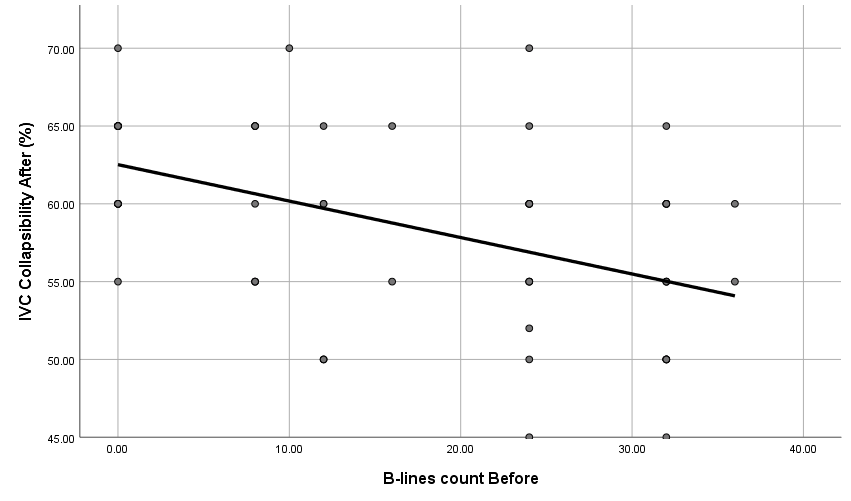 |
| **(e):** Statistically significant negative correlation between B-lines count and IVC collapsibility before HD  with (r-value -0.553 and p<0.001). | **(f):** Statistically significant negative correlation between B-lines count before and IVC collapsibility after HD  with (r-value -0.501 and p<0.001). |
| 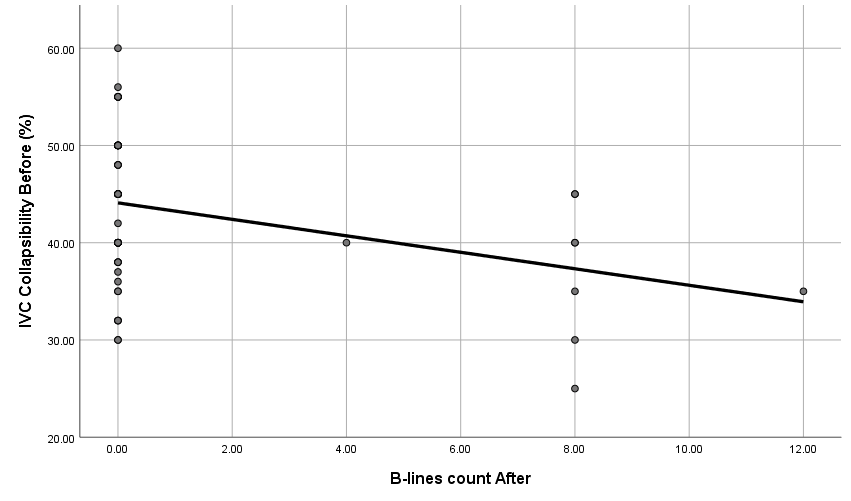 | 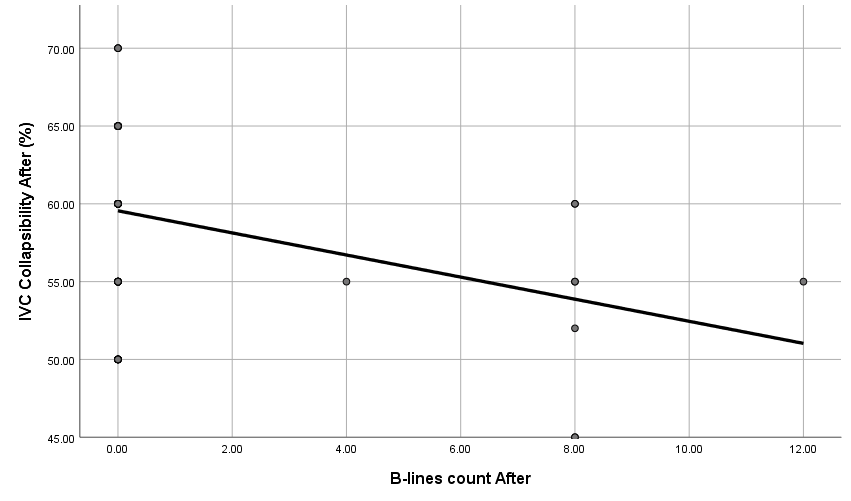 |
| **(g):** Statistically significant negative correlation between B-lines count after and IVC collapsibility before HD with (r-value -0.312 and p=0.015). | **(h):** Statistically significant negative correlation between B-lines count and IVC collapsibility after HD  with (r-value -0.330 and p=0.010). |

**Supp. E: correlation between IVC diameter and collapsibility index, and B-lines count before and after the HD session.**

| 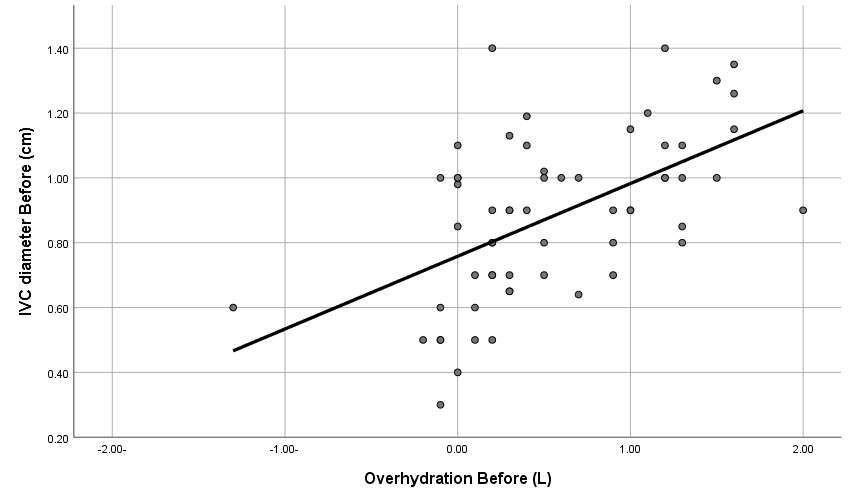 | 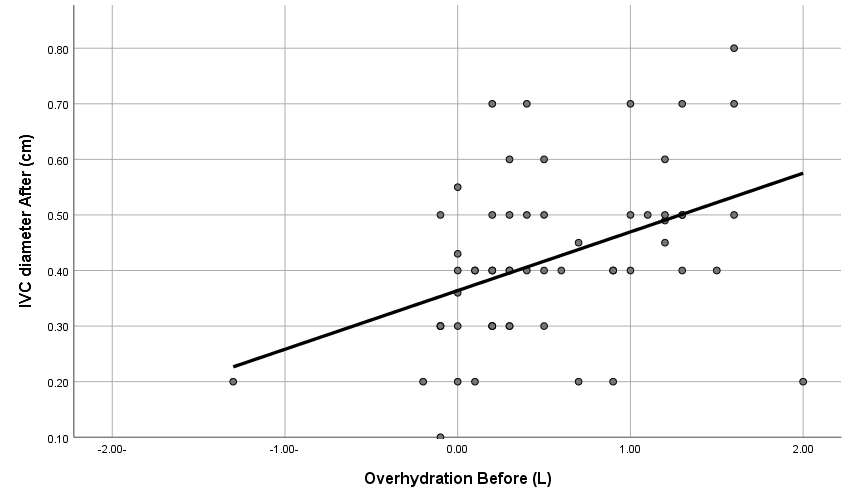 |
| --- | --- |
| **(a):** Statistically significant positive correlation between over hydration and IVC diameter before HD with (r-value = 0.559 and p<0.001). | **(b):** Statistically significant positive correlation between over hydration before HD and IVC diameter after HD with (r-value = 0.461 and p<0.001). |
| 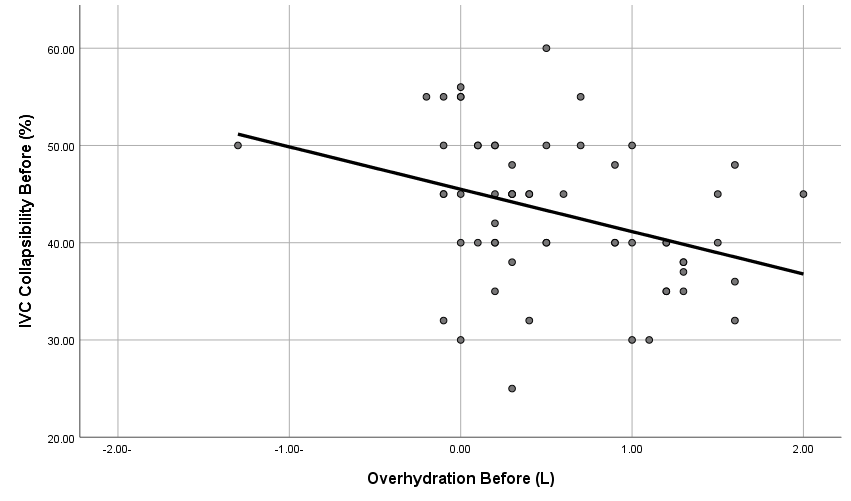 | 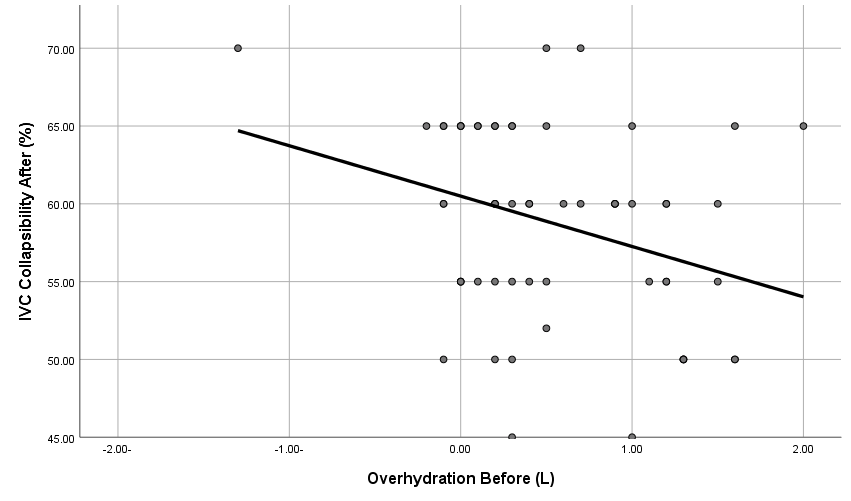 |
| **(c):** Statistically significant negative correlation between overhydration and IVC collapsibility before HD with (r-value -0.391 and p<0.002). | **(d):** Statistically significant negative correlation between over hydration before HD and IVC Collapsibility after HD with (r-value -0.312 and p<0.015). |
| 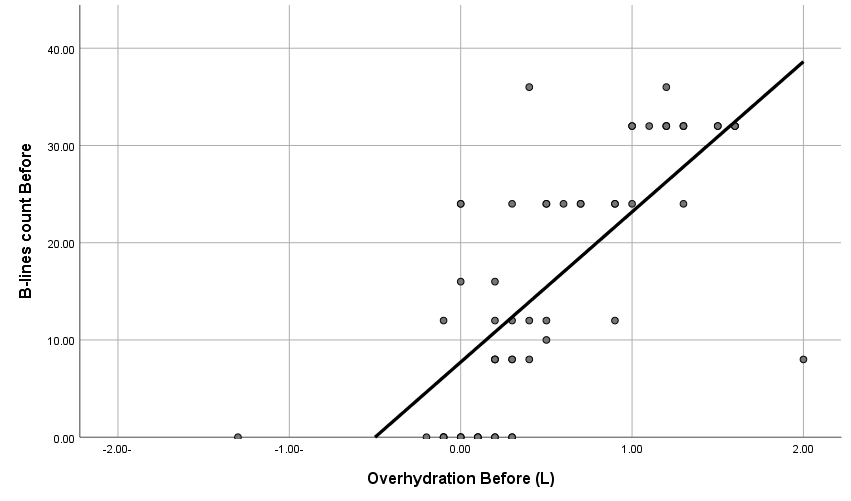 | 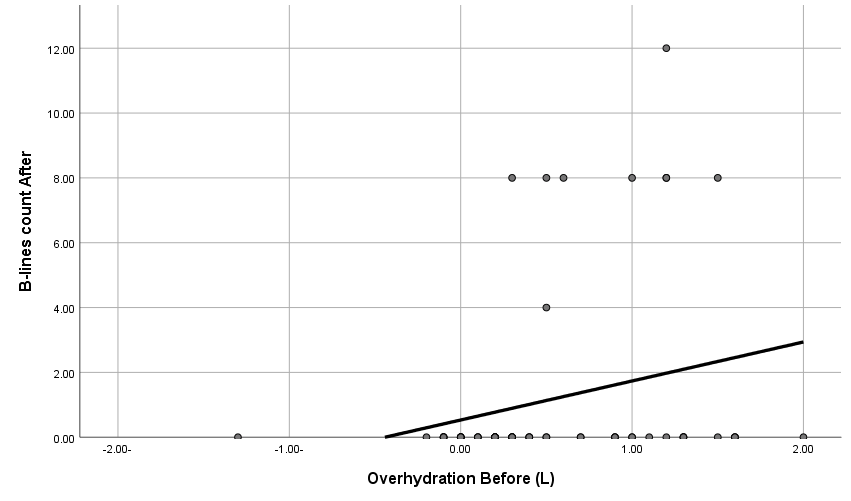 |
| **(e):** Statistically significant positive correlation between overhydration and B-lines count before HD with (r-value = 0.780 and p<0.001). | **(f):** Statistically significant positive correlation between over hydration and B-lines count after HD with (r-value = 0.283 and p=0.029). |

**Supp. F: a significant correlation between the pre-dialysis OH and both IVC parameters (diameter and collapsibility), as well as B-lines count before and after HD.**
